# Using the Community Resilience Model and Project ECHO to Build Resiliency in Direct Support Professionals: Protocol for a Longitudinal Survey

**DOI:** 10.2196/59913

**Published:** 2025-03-06

**Authors:** Kristina Puzino Lenker, Laura L Felix, Sarah Cichy, Erik Lehman, Jeanne M Logan, Michael Murray, Jennifer L Kraschnewski

**Affiliations:** 1 Department of Psychiatry and Behavioral Health Penn State College of Medicine Hershey, PA United States; 2 Department of Internal Medicine Penn State College of Medicine Hershey, PA United States; 3 Department of Public Health Sciences Penn State College of Medicine Hershey, PA United States; 4 Sheppard Pratt Towson, MD United States

**Keywords:** neurodiversity, community resilience model, Project ECHO, direct support professionals, autism, telementoring, methods and feasibility, resiliency, intellectual disabilities, ASD, autism spectrum disorder, DSP, supportive care, community resilience, burnout, resilience, neurodivergent client, neurodevelopmental disorders, evidence-based knowledge

## Abstract

**Background:**

Individuals with intellectual disabilities or autism spectrum disorder (ID/A) sometimes require supportive services from direct support professionals (DSPs). The supportive care provided to individuals with ID/A by DSPs can vary from assistance with daily living activities to navigating society. The COVID-19 pandemic not only exacerbated poor outcomes for individuals with ID/A but also for DSPs, who report experiencing burnout in the aftermath of the pandemic. DSPs are critical to providing much-needed support to individuals with ID/A.

**Objective:**

The goal of this study is to evaluate the impact of the community resilience model on DSP burnout and neurodivergent client outcomes using the Project ECHO (Extension for Community Healthcare Outcomes) telementoring platform as a dissemination tool.

**Methods:**

This protocol leverages community resilience theory and telementoring through the Project ECHO model to foster resilience in DSPs and their neurodiverse client population. ECHO participants’ resilience behaviors will be evaluated via surveys including the Connor Davison Resilience Scale and the WHO-5 Well-Being Index. These surveys will be administered preprogram, at the end of the 8-week ECHO program, and 90 days after the ECHO program’s completion. Pre-post relationships will be assessed using generalized estimating equations. The main outcomes will be self-reported changes in knowledge, self-efficacy, and resilience.

**Results:**

All ECHO program cohorts and follow-up data collection have concluded, with 131 survey participants. The project team is currently analyzing and interpreting the data. We anticipate having all data analyzed and interpreted by February 2025.

**Conclusions:**

DSPs provide critical services to individuals with ID/A. By providing skills in resiliency via the ECHO model, participants will be able to apply resiliency to their own professional lives while fostering resilience within their neurodiverse client base, leading to increased positive outcomes for both groups.

**International Registered Report Identifier (IRRID):**

DERR1-10.2196/59913

## Introduction

Intellectual disabilities and autism spectrum disorder (ID/A) are lifelong, complex neurodevelopmental disorders characterized by social, cognitive, and adaptive skill deficits [1,2]. These skill deficits vary in levels of severity for each individual with ID/A and may require supportive care [2]. Direct support professionals (DSPs) provide supportive care to individuals with ID/A in the form of community integration, including employment navigation, assistance with daily living activities, and advocacy [3].

Individuals with ID/A are especially vulnerable to trauma and may struggle more to identify and implement successful coping strategies. Pairing this with undereducation in recognizing and responding to trauma among the service provider community creates a significant gap in services available to individuals with ID/A. Stress responses in individuals, including those with ID/A, can be moderated by factors that increase resilience. Resilience is defined as a process of interactive adaptation that facilitates coping in the face of adversity linked with a person’s neurological and psychological makeup and socioecological contexts [4]. Fostering resilience for both service providers and individuals with ID/A can enhance their adaptive stress responses across all domains of functioning. Currently, there are limited resources in the community that can adequately address building resiliency, specifically in this population.

Prior to the COVID-19 pandemic, DSPs were already in the midst of a workforce crisis long characterized by low wages, training challenges, and high turnover rates [5]. However, DSPs working with those in the ID/A community reported experiencing lower quality of life and even higher percentages of burnout during the COVID-19 pandemic [6,7]. Evidence suggests that the COVID-19 pandemic exacerbated already-existing support system weaknesses, resulting in the displacement of DSPs that strained an already short-staffed workforce, increased hours worked by those not displaced, and worsened work-life quality [6,8]. These issues, compounded by the complexity of providing supportive care for this population during a global public health crisis, presented the need for resilience training and improved support for DSPs. Resiliency is associated with the achievement of life satisfaction, positive well-being, competent functioning, and improved quality of life after experiencing stressors or adversity [9]. Therefore, fostering resilient behaviors in DSPs as well as their neurodivergent clients is critical to strengthening and empowering neurodivergent individuals and creating more inclusive neurodiverse communities.

To better support individuals with ID/A, DSPs require new and advanced knowledge and skills to provide best-practice care. Providing training and resources in resiliency-building skills would allow DSPs to assist these often-overlooked populations while also providing DSPs with the tools needed to navigate post–COVID-19 working conditions. Further, it is well-known that individuals with ID/A who receive services and support from DSPs experience better personal outcomes, especially when the same DSP is engaged over time [10]. Identifying training and supportive opportunities for DSPs that improve DSP resilience, improve the health and resilience among the ID/A community, and improve the well-being and retention of the DSPs could significantly improve care for adults with ID/A. Unfortunately, current resources and supports that address building resiliency for DSPs are limited. In response to this service gap and the COVID-19 pandemic, Project ECHO (Extension for Community Healthcare Outcomes) at Penn State College of Medicine developed the “Fostering Resilience for Neurodiverse Communities ECHO” program for professionals serving neurodivergent individuals. The goal of this program is to share strategies to build resilience and better support those in their care to address trauma and stress needs as they reintegrate following the COVID-19 pandemic. These strategies were intentionally designed to have wide application so that service professionals could also benefit. The intent was to create resources and strategies that the neurodivergent clients and their service providers could use together in an embrace of more inclusive neurodiverse community building. This protocol aims to detail the Fostering Resilience for Neurodiverse Communities ECHO program conduction, curriculum, data collection, and planned outcomes analysis to facilitate further use of the ECHO model with other audiences, including those supporting neurodiverse populations. Our program’s intended outcome is to invoke positive change in the knowledge, confidence, and resiliency of DSPs after participation in the ECHO program.

## Methods

### Project ECHO

**Figure 1:**
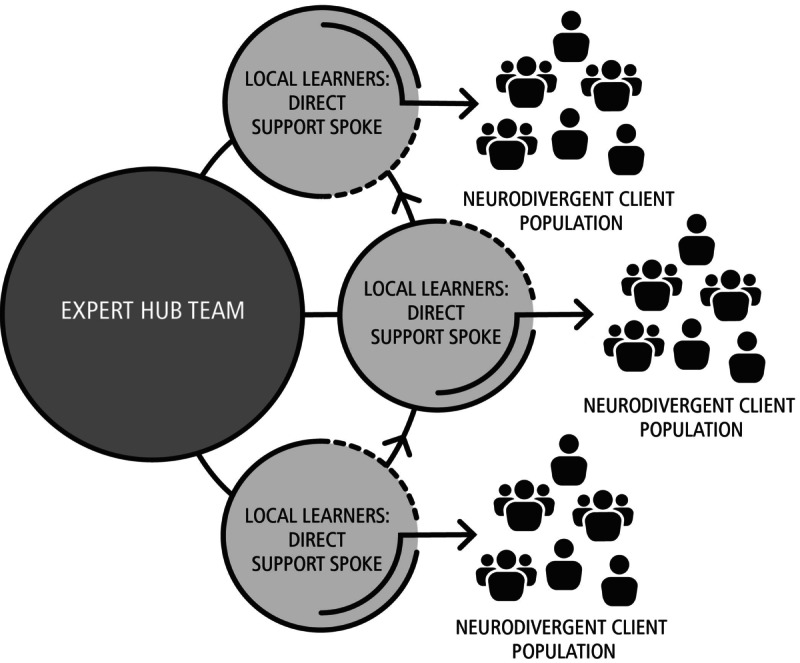
The ECHO (Extension for Community Healthcare Outcomes) hub-and-spoke learning model.

The Fostering Resilience for Neurodiverse Communities ECHO program used the ECHO platform to provide DSPs with evidence-based knowledge and skills to best support their neurodiverse clients in building resiliency following the COVID-19 pandemic. Project ECHO is an evidence-based educational model with the power to rapidly transfer knowledge and exponentially increase capacity to deliver best-practice care to underserved populations [11-18]. The ECHO model’s “all teach, all learn” approach is the infrastructure for knowledge-sharing in underserved communities around the world. The heart of the ECHO model is its hub-and-spoke knowledge-sharing networks, led by expert specialist teams (hub), mentoring multiple DSPs (spokes) via teleconferencing (Figure 1). The ECHO model is not “telemedicine” where specialists assume the care of the client; rather it is a guided model aimed at practice improvement, in which DSPs retain responsibility for clients, and operate with increasing independence as skills, confidence, and self-efficacy grow. Unlike traditional learning, Project ECHO facilitates rapid dissemination of knowledge and increased capacity to deliver best-practice care by utilizing case-based, collaborative learning to support the discussion of learners’ challenges and barriers to guideline implementation.

The ECHO model has 4 core principles: (1) use technology to leverage scarce resources; (2) share best practices to reduce disparities; (3) use case-based learning to master complexity; and (4) monitor outcomes to ensure benefit. DSPs and other professionals (spokes) who serve neurodiverse populations participated in weekly web-based ECHO sessions with a multidisciplinary specialty team at Penn State College of Medicine (hub) with behavioral health, psychiatry, and community resource expertise. Project ECHO used Zoom, a user-friendly, Health Insurance Portability and Accountability Act–compliant, cloud-based software application for video conferencing.

ECHO sessions featured presentations of deidentified client cases by DSPs and team members and brief lectures by specialists. The ECHO specialists and DSPs collaborated to discuss the case and develop recommendations for the DSP and the client. The hub team provided technical assistance and training on resilience building and facilitated the discussion of cases provided by spokes. Together, hub experts collaborated with spoke participants to share knowledge, expertise, best practices, and experiences while discussing deidentified cases and developing recommendations. Over time, spoke participants developed expertise while also engaging in a wider community of learners. Spoke participants were empowered to address the difficulties faced by their clients in their communities. As a result, ID/A clients gained access to best practices and resources through DSP participation in the ECHO sessions.

The Fostering Resilience for Neurodiverse Communities ECHO hub team consisted of multidisciplinary experts who provide care to neurodiverse patients. This team included the Clinical Director and Associate Clinical Director of the Division of Autism Services at Penn State Health Milton S. Hershey Medical Center, a psychologist with experience in behavioral health, a board-certified psychiatric nurse practitioner, a licensed clinical social worker, and a board-certified behavior analyst. Additionally, the expert specialist team also consisted of a neurodivergent self-advocate to provide a client perspective and expertise to case discussions. The project team included a project manager, education specialist, and program director from Project ECHO at Penn State College of Medicine who support the day-to-day operations of the ECHO model.

### Proposed Approach: Community Resilience Model

This protocol details the use of the community resilience model (CRM) to train DSPs to foster resilience in themselves as well as their neurodiverse clients, including individuals with ID/A, through the Project ECHO platform [19]. The overarching objective of the Fostering Resilience for Neurodiverse Communities ECHO series was to teach the skills of the CRM to professionals who serve neurodiverse clients to promote psychological flexibility in themselves and their clients.

The CRM is a community-based approach for coping with acute and chronic stress states [19]. It is a sensory-focused approach aimed at increasing mental well-being and greater resilience, which has been successfully used in diverse populations and settings, including front-line health workers at the height of the COVID-19 pandemic [20,21]. Its neurobiological basis is aimed at down-regulating stress responses in the body through a skills-based approach that has wide applicability. CRM is a community-based approach where community members not only help themselves but also support others within their social networks. CRM aims to create “resiliency-focused communities,” an approach well suited for the intended outcomes of this project [19].

Using CRM, the hub team developed a curriculum addressing trauma, stress, anxiety, improving resilience, and more. A portion of the Fostering Resilience for Neurodiverse Communities ECHO curriculum promoted using the iChill App to teach the CRM through a set of self-help skills [22]. Participants registered for one cohort of the Fostering Resilience for Neurodiverse Communities ECHO program. Each ECHO cohort consisted of 8 weekly 75-minute ECHO sessions. A comprehensive list of topics can be found in Table 1. Goals aligned with the approach are found in Textbox 1.

**Table 1:** Fostering resilience for neurodiverse communities ECHO^a^ series topics.

Session	Didactic topic
1	What is trauma? Traumatic stress, expressions of trauma, and trauma responses
2	Anxiety: Intolerance of uncertainty
3	What is resilience? Introduction to CRM^b^: why is it important? Resilience zone and widening the zone
4	Resilience skills I: Tracking, resourcing, and grounding
5	Resilience skills II: Gesturing, shift and stay, help now!
6	Crisis risk reduction and safety considerations
7	Adapting skills for those with brain differences
8	Fostering community reengagement

^a^ECHO: Extension for Community Healthcare Outcomes.

^b^CRM: community resilience model.

**Intended programmatic goals.**

1. Increased awareness and better recognition of trauma issues related directly to the COVID-19 pandemic and past trauma issues that have been exacerbated by it
2. Better understanding of the community resilience model and the importance of developing greater resiliency for both providers and neurodivergent clients
3. Ability to guide direct support professionals and other team members into effective use of available resources to better meet the needs of the individuals they are supporting

### Participants and Recruitment

Participants for this program were DSPs and those supervising DSPs who participated in Fostering Resilience in Neurodiverse Communities ECHO sessions. Within Pennsylvania, DSPs directly supporting neurodivergent individuals, as well as direct supervisors of DSPs, were recruited through targeted email listservs. An informative recruitment flyer was disseminated directly to DSPs through the Pennsylvania Department of Human Services’ Office of Developmental Programs. The project team also leveraged social media platforms through the autism services, resources, education, and training network to promote the ECHO program.

The initial recruitment survey was distributed via flyer with a public-facing survey link. However, participants were screened after entering basic demographic or contact information, at which point study staff would invite eligible participants to complete questionnaires. Screening criteria included those employed currently as a DSP or as a direct supervisor of DSPs. Once approved by study staff, participants received surveys via email with personalized links, ensuring that all surveys except for the initial recruitment survey were not publicly available. After completion of all preprogram surveys, participants were invited to participate in ECHO sessions.

### Survey Administration

All surveys were hosted electronically and, aside from the public recruitment survey, were only accessible via email. Project data were collected and managed using REDCap (Research Electronic Data Capture), developed by Vanderbilt University and hosted at Penn State Health Milton S. Hershey Medical Center and Penn State College of Medicine, which was supported by Penn State Clinical and Translational Science Institute. REDCap is a secure, web-based application designed to support data capture for research studies [23].

Survey responses were collected across multiple cohorts from August 2022 to August 2024. Initial survey completion was mandatory for participation in the ECHO series, but completion of session evaluations, final program surveys, and follow-up surveys was voluntary.

Survey questions were not randomized and were displayed on a single page per survey. Adaptive questioning was utilized in every survey to minimize survey fatigue. Participants did have the ability to review and change answers before submitting each survey.

Participants answered several demographics (eg, geographic location) and professional questions (eg, credentials, job title, provider type). Participants were also asked about their experience in providing care to neurodiverse populations and how the acuity of care provided to their neurodivergent clients has changed throughout the COVID-19 pandemic. Participants completed several educational evaluations at baseline and postprogram to measure programmatic impact.

The recruitment survey contained 36 questions. The preseries assessment, which was only administered to participants who were screened as eligible for participation by study staff, contained 59 questions. The session evaluations were completed after every ECHO session and contained 14 questions. The final program evaluation contained 83 questions. The 90-day follow-up survey contained 35 questions.

### Response Rates

Survey view rates were not available for this study. Study staff closely monitored response rates. The public recruitment survey was closed once at least 40 eligible participants were enrolled. Study staff screened for eligibility as participants completed the surveys. Additionally, study staff monitored the completion of preprogram questionnaires to ensure that only those who completed the preprogram questionnaires were invited to join the ECHO sessions. Session evaluations, final program evaluations (n=66), and follow-up evaluations (n=51) were also closely monitored by study staff to ensure completion and data integrity.

### Questionnaire Development

Upon registering for a Fostering Resilience for Neurodiverse Communities ECHO cohort, participants answered several demographics (eg, geographic location) and professional questions (eg, credentials, job title, provider type). Participants were asked about their experience in providing care to neurodiverse populations and how the acuity of care provided to their neurodivergent clients has changed through the COVID-19 pandemic.

Participant self-efficacy was measured at baseline and postprogram using adapted versions of measures identified in the literature [22,24]. Respondents completed each item with their respective level of confidence on several items on a 5-point Likert scale ranging from “not confident” to “highly confident.” Next, participants rated their respective level of confidence on a 6-point Likert scale, ranging from “no confidence” to “highly confident” [21,23]. In postprogram evaluation, participants self-reported changes in clinical knowledge and the application and dissemination of knowledge learned from the Fostering Resilience for Neurodiverse Communities ECHO series. This was measured using a series of questions using a 5-point Likert scale, ranging from “strongly disagree” to “strongly agree,” and included “I applied or shared knowledge learned in ECHO sessions to the neurodiverse clients who have ID/A support,” “I applied or shared knowledge learned in ECHO sessions to the direct support professional I supervise,” “I applied or shared knowledge learned in ECHO sessions with my colleagues in similar roles as mine,” “I applied or shared knowledge learned in ECHO sessions with my supervisors and/or agency management team,” and more.

The Connor-Davison Resilience Scale [25] and the WHO-5 Well-Being Index [26] were used to determine the impact on participant resiliency. Participants were asked to report the use of CRM skills. Additionally, participants’ use of the iChill app [22] that is promoted through the ECHO series is explored through a sequence of questions including, “do you use the iChill app on your phone or tablet,” “if yes, how often do you use it,” and “how helpful is the app?” Participants also responded to several questions related to their experience and satisfaction with the Fostering Resilience for Neurodiverse Communities ECHO series.

### Planned Data Analysis

All variables will be summarized using descriptive statistics to assess their distributions and missing data. Pre versus post, pre versus 90-day follow-up, and post versus 90-day follow-up comparisons will be made for all outcome variables. For binary and ordinal (5-category Likert scale) categorical outcomes, we will use binomial or ordinal generalized estimating equation models, which account for the correlation between multiple observations made on each subject over time, to estimate odds ratios between the time points. The magnitude and direction of the odds ratios will allow us to assess the effect size of any significant changes over time. The *P* values for odds ratios that relate to the comparisons between time points, which generally will be all 3 possible comparisons, will be adjusted for multiple comparisons using the Tukey method for each outcome variable. This will maintain a familywise error rate of 0.05. Using this approach will also allow us to include any significant covariates in the model for adjustment if necessary. A sensitivity analysis will be applied to the ordinal outcome variables using quantile regression of the median by using the difference between the outcome responses for each pair of time points as the outcome variable in the model. This will also allow us to adjust for any covariates and use the same Tukey method of adjustment for multiple comparisons. All analyses will be performed using SAS software (version 9.4; IBM Corp) developed by the SAS Institute.

### Ethical Considerations

Approval for this project was obtained from the Penn State Institutional Review Board at the Penn State College of Medicine in Hershey, Pennsylvania (STUDY00018204). Our program was given a not-human subjects research determination because it did not meet the definition of human subject research as defined in 45 CFR 46.102(e) and (l). This determination was made because the intention of this protocol is to assess and improve ECHO processes rather than contribute to generalizable knowledge. Because our program was determined to be a quality improvement activity, consent from ECHO participants was not required. The project data were only accessible to project staff. Participant data were deidentified and aggregated by ECHO staff before use in reporting and analysis.

Monetary incentives were provided for follow-up survey completion for September 2022, January 2023, and April 2023 cohorts only. Monetary incentives were based on the number of surveys completed: completion of at least 7 of 8 session evaluations: US $200; completion of only the 90-day follow-up survey: US $25; and completion of at least 7 of 8 session evaluations AND completion of the 90-day follow-up survey: US $225.

### Reporting Guidelines

We used the Checklist for Reporting Results of Internet E-Surveys (CHERRIES) [24].

## Results

The ECHO cohorts ran from September 2022 to May 2024, with final follow-up survey data collection occurring in August 2024. There were 131 unique participants who attended ECHO sessions across the 6 cohorts. Participants were DSPs or supervisors of DSPs located in Pennsylvania, although 2 participants indicated that they were from other states (New Jersey and Oregon). Participants were from rural (n=35) and urban (n=94) counties, while other participant rurality is unknown (n=2). Data analysis is planned to be concluded by January 2025.

## Discussion

The COVID-19 pandemic has shed further light on the pressing need to provide neurodiverse populations with the skills and resources necessary to successfully navigate society and the ongoing workforce crisis impacting the DSPs serving these populations. In response to the need presented to equip DSPs with resilience-building tools for themselves and their neurodivergent clients, Project ECHO at Penn State College of Medicine launched the Fostering Resilience for Neurodiverse Communities ECHO series. This ECHO series trains DSPs in ways to foster resilience in their neurodivergent clients and themselves. We anticipate that DSPs participating in the ECHO program will report feeling more confident and resilient in their roles.

The CRM has been implemented in other audiences, including health care professionals such as nurses, and it was found that brief resiliency trainings using CRMs for nurses resulted in improved well-being and resiliency among participants [25]. Additionally, the Project ECHO model has been used in resiliency training for first responders, resulting in increased confidence in resiliency skills [26]. The application of the CRM paired with the Project ECHO model for DSPs outlined in this protocol is a novel approach to resiliency training. Other applications of the CRM occur over shorter periods, with one training session lasting 3 hours [21,25]. The application of the CRM in this protocol will give DSPs tools to apply CRM principles in 75-minute ECHO sessions, held once per week over 8 weeks, leveraging active learning, engagement, and community-building, which defines the Project ECHO model. While our study is innovative and comprehensive, it is not without limitations. We rely on self-reported measures of knowledge acquisition, self-efficacy, resiliency building, and satisfaction, potentially resulting in self-report bias of over or underreporting a change in the above-mentioned outcomes. Other less biased outcome measures include supervisor-reported questionnaires, usage data of the iChill application, and retention rates of DSPs, which can be incorporated into future studies.

The Fostering Resilience for Neurodiverse Communities ECHO program introduces a unique opportunity for the application of resilience-building strategies across a variety of contexts to individuals who can apply them directly to their work and in real time. Common themes already identified within the ongoing ECHO program indicate the need for more trauma-informed training among DSPs and other professionals who serve neurodiverse populations, particularly in underserved and marginalized communities. We anticipate this training will result in increased self-efficacy, improved well-being, and expanded application of resiliency skills for DSPs and their neurodiverse clients.

## Abbreviations

CRM: community resilience model
DSPs: direct support professionals
ECHO: Extension for Community Healthcare Outcomes
ID/A: intellectual disabilities and autism spectrum disorders
REDCap: Research Electronic Data Capture
